# Non-Canonical Localization of Cardiac Troponins: Expanding Functions or Causing Pathologies?

**DOI:** 10.3390/ijms25063117

**Published:** 2024-03-08

**Authors:** Eugene A. Arifulin, Eugene V. Sheval

**Affiliations:** 1 Belozersky Institute of Physico-Chemical Biology, Lomonosov Moscow State University, 119991 Moscow, Russia; sheval_e@belozersky.msu.ru; 2 Department of Cell Biology and Histology, School of Biology, Lomonosov Moscow State University, 119991 Moscow, Russia

**Keywords:** cardiac troponin, nuclear localization, importin, transcription, NLS, NoLS, mitochondrial localization, extracellular troponins

## Abstract

The troponin complex—consisting of three subunits: troponin C (TnC), cardiac troponin I (cTnI) and cardiac troponin T (cTnT)—plays a key role in the regulation of myocardial contraction. Troponins are preferentially localized in the cytoplasm and bind to myofibrils. However, numerous, albeit scattered, studies have shown the presence of troponins in the nuclei of muscle cells. There is increasing evidence that the nuclear localization of troponins may be functionally important, making troponins an important nuclear player in the pathogenesis of various diseases including cancer and myopathies. Further studies in this area could potentially lead to the development of treatments for certain pathologies. In this review, we collected and discussed recent data on the properties of non-canonically localized cardiac troponins, the molecular mechanisms leading to this non-canonical localization, and the possible functions or pathological effects of these non-canonically localized troponins.

## 1. Introduction

A canonical muscle cell contractile unit—the sarcomere—contains bundles of properly arranged actin and myosin filaments, whose interactions are regulated by accessory proteins, troponins (Tns), and tropomyosin (Tm). The troponin-tropomyosin complex utilizes changes in intracellular Ca^2+^ concentration to generate muscle contraction [1]. The troponin complex consists of the Ca^2+^-binding subunit—troponin C (TnC), tropomyosin-binding subunit—troponin T (TnT), and inhibitory subunit—troponin I (TnI). Each Tn subunit has several forms that are specific to skeletal muscle, cardiac muscle, or different muscle variants [2]. TnC has two known forms: a slow TnC isoform (TnC, gene *TNNC1*) that is specific for both skeletal and cardiac muscles and a fast skeletal TnC isoform (fsTnC, gene *TNNC2*). TnT has three forms: slow skeletal (ssTnT, gene *TNNT1*), fast skeletal (fsTnT, gene *TNNT3*), and cardiac (cTnT, gene *TNNT2*). TnI also exists in three different forms: slow skeletal (ssTnI, gene *TNNI1*), fast skeletal (fsTnI, gene *TNNI2*), and cardiac (cTnI, gene *TNNI3*).

The study of cardiac Tns (cTns) is important, not only for identifying the molecular mechanisms of muscle contraction. After myocardial infarction, intracellular proteins from dying cardiomyocytes are released into the bloodstream, and several proteins (e.g., myosin light chains [3] and cTns [4]) can be identified in the blood of patients with myocardial infarction. TnI and TnT are exclusively present as specific cardiac isoforms (cTnI and cTnT, respectively) in cardiomyocytes, which allows the use of Tn-based assays for acute myocardial injury in routine clinical practice [5].

For many years, Tns have been considered to be “typical” cytoplasmic proteins associated with the myofibrils of differentiated muscle cells and specialized to perform a unique function. Back in 2001 Kheyat et al. demonstrated that embryonic stem cells can differentiate into cardiomyocytes which provided a suitable model for in vitro studying of heart development [6]. Numerous studies in recent decades have shown that Tns are at least partially localized in the nuclei of muscle cells [7,8,9,10,11,12]. The first published observation of intranuclear cTn was described in 2009 by Bergman et al., who used this protein as a marker to isolate cardiomyocyte nuclei by fluorescent sorting [7,9]. Around the same time, Sahota et al. found Tn in the nuclei of *Drosophila* S2 cells, suggesting that it plays an important role in maintaining nuclear integrity [8]. A detailed study of nuclear cTn and other motor proteins was performed in 2012, when Asumda et al. established their role in Ca^2+^ level modulation [10]. The authors proposed that these proteins could form a complex structure inside the nucleus and retain Ca^2+^ ions, as they do in the cytoplasm. Two years later, Chen et al. described the presence of cTn in non-muscle cells such as non-small cell lung cancer tissue and cancer cell lines, suggesting its role as a cancer diagnostic marker [11]. In 2019, Kharitonov et al. discovered potential nuclear localization signals (NLSs) and nucleolar localization signals (NoLSs) in the primary sequence of cTnI and proposed the mechanism of its nuclear accumulation [12]. Thus, some authors speculate that this protein may have specific regulatory functions in addition to its canonical role as a regulator of actin-myosin interactions (for review see the work presented in [13]). In this review, we discuss recent new data on the possible nuclear functions of Tns, possible mechanisms of their nuclear localization (with special attention to cTns), and the possible impact of such localization. Available data suggests that the nuclear localization of Tns may be both a part of the normal functional mechanisms of the cell and an element in the development of pathological processes.

## 2. Role of Cardiac Troponins in the Epigenetic Regulation of Gene Expression

Over the past decade, several studies revealed that cTn subunits are implicated in epigenetic regulation through their interactions with histone-modifying enzymes. Therefore, some pathogenic cTn mutations previously thought to impair sarcomeric contractility may also affect their nuclear function.

For example, cTnI is likely to be involved in epigenetic regulation, because it interacts with histone deacetylase 1 (HDAC1) and SET and MYND domain containing 1 (SMYD1), as shown by co-immunoprecipitation [14]. Overexpression of mutant cTnI193His in transgenic mice results in decreased expression of phosphodiesterase 4D (PDE4D), which is regulated by HDAC1 and SMYD1 [14]. In a subsequent study, Zhao et al. (2021) confirmed these results by overexpressing HDAC1 in cultured primary cardiomyocytes, which led to a reduction in PDE4D. The same reduction in PDE4D mRNA and protein levels was achieved by the overexpression of cTnI193His in cultured primary cardiomyocytes. The authors suggested that cTnIR193His may downregulate PDE4D via HDAC1-induced deacetylation of H3K4 and H3K9 in PDE4D promoter regions [15] It is not completely clear how cTnIR193His affects HDAC1 binding to the PDE4D promoter region, but the mutant version of cTnI shows a stronger affinity for HDAC1 than the wild-type cTnI. The authors showed that epigallocatechin gallate (EGCG) alleviated the reduction in PDE4D induced by the cTnIR193H mutant but had no effect on HDAC1 expression and activity. In contrast, the strength of the interaction between cTnIR193H and HDAC1 decreases after EGCG treatment [15].

Similar to cTnI, the cTnT subunit was also found to interact with histone-modifying enzymes, such as lysine (K)-specific demethylase 1A (KDM1A) and lysine-specific demethylase 5A (KDM5A). Wu et al. used induced pluripotent stem cells (iPSCs) from patients with dilated cardiomyopathy (DCM) to study the cellular mechanisms of DCM pathogenesis [16]. Mutated cTnT is more likely to be located in the nuclei of DCM iPSC cardiomyocytes (iPSC-CMs) than in nuclei of control iPSC-CMs. The cTnTR173W mutation, which is associated with DCM, appeared to increase the nuclear accumulation of cTnT and enhance its interaction with KDM1A and KDM5A. The authors suggested that in DCM cardiomyocytes, such interactions may affect the distribution and activity of histone demethylases, resulting in the increased expression of active epigenetic markers in *PDE2A* and *PDE3A* genes [16]. Importantly, according to these data, TnT is involved in the epigenetic control of PDE expression in the nucleus; thus, the mutation affects cTnT function not only in the myofilament lattice, but also in the nucleus.

## 3. Cardiac Troponins and Ca^2+^-Regulation

cTnI dysfunction can lead to various heart diseases, such as DCM, hypertrophic cardiomyopathy (HCM), and restrictive cardiomyopathy (RCM) in humans [17,18,19,20,21,22,23]. It has been suggested that these disorders may be caused by loss of cTn intranuclear activity rather than its canonical cytoplasmic function [24]. According to published data, an important aspect of the effect of cTn on nuclear processes is the modulation of Ca^2+^ level [10]. Recent studies have shown that cTnI may regulate the *Atp2a2* gene, which encodes sarcoplasmic/endoplasmic reticulum Ca2+ ATPase 2a (SERCA2a) [25]. Experiments in a knockout mouse model have shown that cTnI and SERCA2a have a linear correlation of their expression. The chromatin immunoprecipitation sequencing (ChIP-Seq) revealed a cTnI binding target motif “CCAT” enriched in the promoter of the *Atp2a2* gene. This motif is also a binding target for the Yin Yang 1 (YY1) regulatory protein [26,27], which has been shown to interact with cTnI. Summarizing these data, authors suppose that cTnI regulates *Atp2a2* gene activity by interacting with YY1 [25]. It should be noted that, although YY1 suppresses transcription of fetal mouse ssTnI, it has no significant effect on cTnI expression in postnatal hearts [27].

Five proteins involved in muscle contractility are present in the nuclei of differentiating cardiomyocytes: all subunits of cardiac troponin (cTn), cardiac tropomyosin (cTm) and actin [10]. Only actin was found in the nuclei of undifferentiated multipotent rat cells, whereas other proteins were observed as early as five days after differentiation induction. The authors suggested that these components could potentially assemble into a structure similar to the cytoplasmic actin-cTn–cTm complex and participate in Ca^2+^ regulation [10].

## 4. Mechanism of Nuclear Accumulation of Cardiac Troponins

Most Tn localization studies have focused on embryonic stem cells or mature myocytes [6,8,10]; however, these models are not perfectly suited for studying the molecular mechanisms of protein trafficking between the nucleus and cytoplasm. However, we know, that exogenously expressed cTnI fused to EGFP is partially localized in the nuclei of several non-muscle cell types [12]. This localization is not an artifact, as similar localization has been observed for endogenous cTns. Indeed, they were expressed in several human cancer cell lines, and immunocytochemistry images obtained from the Human Protein Atlas database showed that cTnC was localized in the nucleoplasm of cervical carcinoma (HeLa), hepatocellular carcinoma (HepG2), and osteosarcoma (U2OS) cells [13]. Similarly, cTnT accumulated in the nuclei and nucleoli of epidermoid carcinoma (A-431), rhabdomyosarcoma (RH-30) and U2OS cell lines [13]. Thus, EGFP-fused cTn is a suitable model for studying the nuclear import of cTn.

According to bioinformatics analysis, cTnI contains NLSs and can potentially be transported through the nuclear envelope via the classical importin-α/β-dependent pathway [7,12]. Although it may seem illogical at first, there are many cytosolic proteins in which NLSs can be predicted [12]. Six “classical” NLSs were also predicted for cTnI, some of which overlapped. Importantly, all the predicted NLSs were located within the conserved protein regions. According to site-directed mutagenesis data, all the predicted NLSs affected the nuclear accumulation of cTnI, demonstrating their common effect. Furthermore, cTnI was partially localized to the cytoplasm in the presence of a peptide inhibitor of importin-α (Bimax2). Simultaneously, cTnI was shown to shuttle freely throughout the nuclear envelope, as expected, because it is a relatively small protein (human cTnI-24kDa). It appears that an importin-α-dependent mechanism led to the nuclear accumulation of cTnI, but free diffusion through nuclear pore complexes limited this accumulation.

After myoblasts differentiate into mature muscle cells, cTnI re-localizes into the cytoplasm [12]. This process appears to be driven solely by myofibril formation, which serves as a retention depot for cTnI molecules and leads to a decrease in the nuclear cTnI fraction.

Importantly, nuclear cTnI also accumulates in the nucleoli. Nucleolar accumulation of some proteins depends on the presence of short motifs called NoLSs [28]. It has been shown that NoLS are enriched in positively charged amino acids and accumulate in the nucleoli due to electrostatic interactions with nucleolar components [29,30,31,32]. Unfortunately, the consequences of nucleolar accumulation remain unclear.

**Figure 1:**
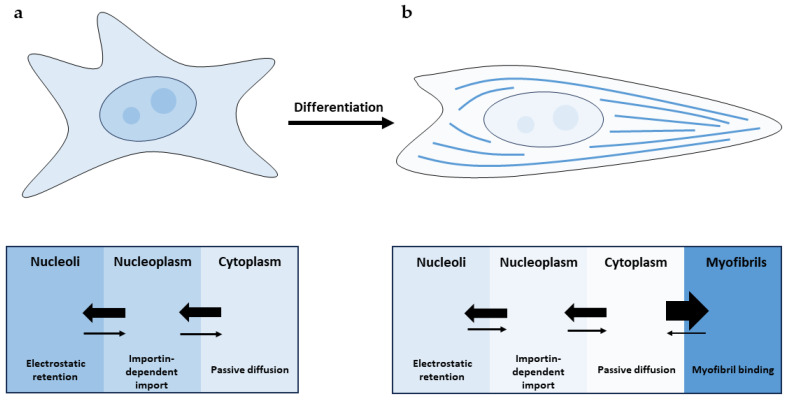
Cardiac troponin I (cTnI) distribution in undifferentiated (**a**) and muscle (**b**) cells. (**a**) cTnI enters the nucleus via importin-dependent import and partially accumulates in the nucleoli via electrostatic interaction with RNA. Diffusion through the nuclear pore complex leads to decreased nuclear accumulation. As a result, cTnI is distributed throughout the cell. (**b**) Concentration of unbound cTnI decreases in all compartments since it binds to myofilaments with high affinity.

Finally, the question of whether Tns can be found in the nuclei of normally differentiated cardiomyocytes should be discussed. Using both paraffin sections and flow cytometry, it has been demonstrated that cTn subunits persist in all cardiomyocyte nuclei in the heart [9]. This is possible if not all Tns are bound to myofibrils. Although both cTnI and cTnT are predominantly bound to myofibrils of cardiomyocytes, significant amounts (5–10%) of both cTnI and cTnT are also found in the unbound cytosolic form [33,34]. Apparently, three fractions coexist in cardiomyocytes: one bound to myofibrils (predominant in differentiated cells) and two unbound fractions (nuclear and cytosolic) that are most likely to be in constant exchange with each other (Figure 1).

## 5. Skeletal Troponins Can Also Modify Nuclear Processes

The main difference between the cardiac and nuclear isoforms lies in the TnC subunit, as the cardiac isoform binds one Ca^2+^ ion, whereas the skeletal isoform binds two. As a result cTn shows lower Ca^2+^ sensitivity than sTn. In addition, cTnC binds with a lower affinity to cTnI than the skeletal isoform. Although the kinetics of Tn interaction with common partners differ between isoforms, the general mechanism remains the same. Since different variants of Tns are quite similar to each other [2], it is logical to expect that skeletal Tns will behave similarly to cardiac Tn. Indeed, fsTnT has been shown to accumulate in the nucleus and be involved in transcriptional regulation [35]. The authors demonstrated that fsTnT, as well as its C- and N-terminal regions, are localized in the nuclei of myoblasts (C2C12), fibroblasts (NIH3T3), and differentiated myofibers. The C-terminal region of fsTnT accumulated in the nucleoli and colocalized with fibrillarin, whereas the N-terminal region tended to localize in the cytoplasm. Overexpression of full-length fsTnT in C2C12 cells resulted in translocation of fibrillarin to the nucleolar periphery. The authors suggested that the expression of fsTnT and its C-terminal region in C2C12 cells caused abnormal cell morphology, similar to fibrillarin depletion. Importantly, both fsTnT and its C-terminal region attracted polymerase I to the nucleoli, whereas only the full-length protein attracted polymerase II. Although the authors used engineered fragments of fsTnT in their study, they found endogenous C- and N-terminal regions in the lysed nuclear fraction of old (26–28 months) mice. This was accompanied by an overall decrease in fsTnT nuclear fraction with age. These data suggest that fsTnT is likely involved in transcriptional regulation, whereas its components appear in old cells and can disrupt their morphology and function [35]. Another study showed that fsTn directly regulates the expression of calcium channel, voltage-dependent, L type, alpha 1S subunit (CACNA1S, Ca_v_1.1) [36]. While investigating how fsTn accumulates and functions in the nucleus, the authors identified an NLS/NoLS sequence at its C-terminus. They also identified a leucine zipper domain in the C-terminal region, which is known to regulate transcription factor binding to DNA. Excision of this motif had no effect on nuclear accumulation but significantly reduced the cytotoxic effect of fsTn overexpression [37]. This result agrees well with previous studies demonstrating high cytotoxicity of the C-terminal and mid-regions of fsTn [38]. The medical significance of these studies is that the cytotoxic effects of free fsTn and/or its components are a probable cause of age-related sarcopenia. [35,37].

Important results have been obtained in *Drosophila*, where the troponin-tropomyosin complex contains TnI and two tropomyosins (Tm1 and Tm2) that are thought to form heterodimers. In S2 cell line cultures, native TnI was found in the nucleus and immunoprecipitated from nuclear extracts [8]. The TnI protein sequence revealed no obvious nuclear localization signal, and SUMOylation of a sequence in exon 10 of TnI is required for nuclear translocation. The authors proposed that the troponin–tropomyosin complex functions as a regulator of the motor systems required to maintain nuclear integrity and apicobasal polarity during early *Drosophila* embryogenesis [8].

Using co-immunoprecipitation, fsTnI was shown to interact with estrogen receptors in the human mammary gland. It increases the transactivity of estrogen-related receptor alpha (ERRα) and, subsequently, ERRα-mediated transcription [39].

The examples presented here show that skeletal Tns can influence different nuclear processes, suggesting that all Tns may be involved in non-canonical functions.

## 6. Other Cytoskeleton Proteins Also Localize and Function in the Nucleus

Nuclear localization of proteins that are traditionally considered to be cytoplasmic is not a rare event, and it is not surprising that several cytoskeletal proteins are not exclusively but partially nuclear-localized and that their nuclear presence is essential for the cell [40,41,42]. The nuclear localization and functions of actin have been extensively studied. Actin is an important cytoskeletal protein that, together with the motor proteins myosins, plays a key role in cell motility, including muscle contraction.

Actins and myosins are involved in various nuclear processes. In particular, actin has been linked to many processes that regulate gene expression [43,44,45,46]. Actin interacts with essentially all transcribed genes in *Drosophila* ovaries [47], copurifies with all three eukaryotic RNA polymerases [44,45,48], and regulates the activity of specific transcription factors [49]. In addition to gene expression, actin is linked to DNA replication [50], DNA damage response [51,52,53,54,55], and long-range chromatin motion [56,57,58].

As well as actin, nuclear myosins are also required for transcription [45,59,60]. Particularly, it was directly shown that myosin VI in the nucleus acts as the molecular anchor that holds RNA polymerase II (RNAPII) in high density clusters, and inactivation or suppression of myosin VI expression leads to changes in RNAPII localization and general chromatin rearrangement [61].

Monomeric G-actin constantly shuttles between the cytoplasm and nucleus [62]. Nuclear export depends on binding to exportin 6 [62,63,64]. According to several reports, the nuclear import of actin depends on importin 9 [47,62,65]; however, a recent study indicated that multiple importins can transport actin into the nucleus in *Drosophila* [66]. Interestingly, actin, which has no NLS, is imported into the nucleus in complex with cofilin [67], which contains NLS [68,69].

Several myosins have been found to accumulate inside nucleoli [70,71]. The mechanisms of nuclear-cytoplasmic transport have been the best studied for myosin IC. Myosin IC has an NLS sequence in the neck region, and importin β, importin 5 and importin 7 were identified as putative nuclear transport receptors which are necessary for nuclear import [72]. Calcium ions play the most important role in regulating the intracellular localization of myosin IC, the elevation of which leads to the activation of myosin IC import into the nucleus [73]. Simultaneously, calmodulin, which binds to the neck region of myosin IC [74], inhibits nuclear transport of the protein [72]. It appears that the elevation in intracellular calcium concentration causes the dissociation of calmodulin from myosin IC and stimulates the transport of this protein into the nucleus, probably because of the exposure of the NLS necessary for binding to importins [73]. Importantly, some data indicate that myosin IC can use a principally different phosphoinositide-dependent pathway for nuclear localization [75]. Myosins VI and XVI appear to be transported to the nucleus via a canonical mechanism involving the NLS [48,61]. However, the exact molecular mechanisms of cytoskeletal protein nuclear-cytoplasmic trafficking require further study.

Thus, the data obtained suggests that nuclear localization is common for cytoskeletal proteins. Interestingly, the mechanisms of accumulation may be different: either the presence of intrinsic NLS or interaction with proteins possessing intrinsic NLS. We analyzed the presence of NLS among cytoplasmic proteins and showed that the proportion of cytosolic proteins with predicted NLS is relatively high (about 50% of all proteins), and these cytoplasmic proteins can potentially accumulate in nuclei [12].

## 7. Mitochondrial Localization of cTnI

Numerous studies have provided evidence that cTnI mutations such as cTnI R193H or cTnI G203S affect mitochondrial structure and activity [76,77,78]. In a recent preprint, Elezaby et al. [79] reported that cTnI localizes in the mitochondrial matrix of the rat heart, rat cardiac myoblasts, and HEK cells. Expression of cTnI in non-cardiac HEK cells results in the suppression of mitochondrial activity, as manifested by impaired oxidative phosphorylation, a 30% decrease in ATP levels, a decrease in mitochondrial membrane potential, and increased sensitivity to oxidative stress induced by H_2_O_2_ treatment. Using a proximity ligation assay, the authors demonstrated that cTnI interacts with the F1F0 ATP synthase subunit D. The F1F0 ATP synthase complex catalyzes both ATP synthesis under normal conditions and ATP hydrolysis during hypoxia. This complex is also a key component of the mitochondrial permeability transition pore (mPTP) opening during cell stress. A protein–protein interaction between cTnI and ATP synthase subunit α (ATP5f1a) has been previously observed in a cross-linked mouse heart proteomics dataset [80]. In vitro experiments showed that the cTnI-ATP synthase interaction resulted in decreased ATP synthesis and increased mPTP opening in response to H2O2 treatment. Apparently, the N-terminus of cTnI plays a key role in this interaction, since mutant proteins, as well as ssTnI, which lacks the N-terminus, did not affect ATP synthase activity. The authors suggested that cTnI may inhibit ATP synthase and mitochondrial functions in respiring mitochondria (under basal conditions), increase ATP hydrolysis under hypoxic conditions, and affect the stability of the ATP synthase complex under stress conditions [79]. It should be noted that so far, the authors have only published a preprint version of their work, so further investigations and revisions in this area are needed.

## 8. Extracellular Cardiac Troponins

It has long been known that in certain diseases (e.g., myocardial infarction), significant concentrations of Tns can be detected in the blood serum; clinical measurement of cTn levels began in the 1990s. Currently, cTnI and cTnT are the preferred biomarkers for both ruling in and ruling out myocardial injury, and thus, for the detection of myocardial infarction [5,81,82]. Various factors have been suggested for the release of Tns from the myocardium, including normal turnover of cardiomyocytes, release from cardiomyocytes via vesicular transport, cellular release of cTn degradation products, increased cellular membrane permeability, cardiomyocyte necrosis, and/or apoptosis [83]. The introduction of extremely sensitive cTn assays (“high sensitivity cardiac troponins”-hs-cTnI and hs-cTnT) has led to increased recognition of myocardial injury in different illnesses. Moreover, cTn molecules were detected in the blood serum of almost all cardio-healthy people [84,85].

The normal presence in serum raises the question of the possible effects of extracellular Tns, and some experimental data indicate that serum Tns could influence various processes. For example, immunization of mice with recombinant murine cardiac troponin I (mc-TnI) resulted in severe myocardial inflammation with increased expression of inflammatory chemokines and chemokine receptors [86]. This inflammation is followed by fibrosis and heart failure, resulting in increased mortality in mice. In contrast, mice immunized with murine cardiac troponin T (mc-TnT) showed little to no inflammation and no death. Myocarditis can result from various infectious and noninfectious causes, including autoimmune responses to cardiac antigens. Various intracellular cardiac antigens, such as cardiac myosin heavy chain α [87,88,89], cTnI [86,90], and adenine nucleotide translocator 1 (ANT1) [91], have been identified as autoantigens in cardiac autoimmunity. It is possible that cTnT-mediated autoimmune response may lead to age-related loss of muscle mass and strength (sarcopenia) [92].

## 9. Cardiac Troponins Inside Non-Cardiac Cells

The examples of Tn localization in the nucleus of undifferentiated cells described above may be a special case if they are expressed not only in cardiomyocytes [10]. Several independent research groups have used polymerase chain reaction and Western blotting to detect cTnT and cTnT in the skeletal muscles of patients with end-stage chronic kidney failure [93]. It has been suggested that an increase in cTn may be due to reduced renal cTn clearance [94]. cTnT expression has been observed in the skeletal muscles of patients with various hereditary myopathies, with no evidence of cardiac disease [95,96]. Importantly, elevated serum levels of cTns were detected in these patients, although there was no clear evidence of cardiovascular disease. However, these data were not confirmed in an independent study [97]. The authors of the latter study found that the cross-reactivity of the cTnT immunoassay with skeletal muscle Tn isoforms may be the cause of this effect. Therefore, the possibility that cTn is expressed in some non-cardiomyocytes cannot be excluded at this time, but should be considered with caution. This problem can most likely be solved by reanalyzing accumulated high-throughput RNA sequencing data.

However, there is no doubt that various Tns, including cTns, can be expressed in nonmuscle cells. The expression of some Tn genes has also been observed in different non-muscle cells, including the human corneal epithelium [98], brain [99,100,101], lung [102], liver [100], and endothelial cells of the rat brain after stereotactic radiosurgery [103]. In addition, Tns are expressed in different cancers [11,13,104]. In particular, cTnI protein has been found in human non-small cell lung cancer tissue and cancer cell lines [11], cTnT in colorectal cancer [105,106]. Interestingly, cTnT promotes the proliferation, invasion, and metastasis of colorectal cancer cells [105]; however, analysis of cTnT localization in this study was not performed. In lung adenocarcinoma, the expression of TnC is strongly downregulated compared to that in normal lung tissues, and downregulation of TnC is strongly correlated with increased mortality [102].

## 10. Conclusions

In recent years, new evidence has emerged demonstrating that the nuclear localization of Tns is not an exceptional event. Even in differentiated cells, some Tns were found in the fraction not bound to myofibrils; this protein can diffuse into the nuclei. The possibility of active transport into the nucleus, previously assumed based only on NLS predictions, has now been experimentally demonstrated in detail, at least for cTnI [12]. Thus, there is increasing evidence that Tn plays an important role in cell function. At the same time, nuclear localization can also lead to the development of pathological processes, either as a disease-causing factor or as a disease-promoting factor. Nuclear Tns may both extend the normal functions of Tns and contribute to the development of various pathologies.

cTn is a well-known clinical biomarker of cardiac injury. However, its involvement in the development of age-related sarcopenia has only been established in the last decade, and further studies in this area could potentially lead to treatments that would slow this process and prolong the life of the heart. Another promising observation is the expression of cTn in nonmuscle cells. This could serve as a potent biomarker of cancerous tissue and a tool for targeted therapy. Some Tn mutations could potentially alter its binding affinity to actin and Tm resulting in increased level of unbound protein affecting its intranuclear concentration. However, we have not found any publications establishing such interconnections. It should be noted that the data describing Tn localization and non-canonical function is rather scarce. Tns are well-studied proteins with respect to muscle contraction and clinical aspects. Numerous studies describe the Tn mutations that cause various muscle pathologies. However, only a few publications provide reliable data on the nuclear functions of Tn. Although the existence of an intranuclear Tn fraction is known, the current picture is still incomplete and further research may lead to the discovery of new Tn functions and interactions in other cellular compartments as well as outside of the cell.

## Data Availability

No data were used for the research described in the article. Data sharing is not applicable to this article.
